# Automating benefits delivery: lowering health insurance costs for unemployment insurance recipients

**DOI:** 10.1093/haschl/qxae054

**Published:** 2024-05-02

**Authors:** Langou Lian, Marina Lovchikova, Andrew Feher

**Affiliations:** California Department of Health Care Access and Information, Sacramento, CA 95833, United States; Covered California, Sacramento, CA 95815, United States; California Department of Health Care Access and Information, Sacramento, CA 95833, United States

**Keywords:** health insurance affordability, automating benefits delivery, American Rescue Plan Act

## Abstract

To provide financial relief to those affected by the COVID-19 pandemic, from July to December 2021, the American Rescue Plan Act temporarily expanded eligibility for cost-sharing reduction (CSR) silver 94 plans that cover 94% of medical costs for unemployment insurance (UI) recipients enrolled in the Affordable Care Act (ACA) Marketplaces. In June 2021, California's ACA Marketplace automatically redetermined eligibility and enrollment for 79 645 UI recipients so the enhanced subsidies would be applied without any action required among program participants. Using administrative data from California and a difference-in-differences design, we found that enrollees automatically moved to CSR silver 94 plans for the second half of 2021 saved $295 in premiums and $180 in out-of-pocket expenses (or $475 in total). These findings can inform state and federal policymakers exploring ways of automating benefits delivery for consumers already engaging with other safety-net programs to increase health insurance affordability.

## Introduction

The Affordable Care Act (ACA) transformed the individual market through the provision of income-adjusted advanced premium tax credits to reduce monthly premiums and through cost-sharing reduction (CSR) subsidies to reduce out-of-pocket costs for low-income consumers who enroll in silver tier coverage. These CSR silver plans increase the coverage generosity of the base silver 70 plan and include 3 variants: CSR silver 73 plans that cover 73% of medical costs for those with incomes between 200% and 250% of the Federal Poverty Level (FPL), CSR silver 87 plans that cover 87% of medical costs for those with incomes between 150% and 200% of the FPL, and CSR silver 94 plans that cover 94% of medical costs for those with incomes below 150% of the FPL.

Amid the COVID-19 pandemic, the US government passed the American Rescue Plan Act (ARPA) of 2021, which funded over 200 programs designed to counteract the pandemic's adverse effects, including enhancing the ACA's subsidy structure. Specifically, the ARPA (1) lowered the required contribution percentage for all ACA enrollees, (2) capped premium contributions at 8.5% of income for those above 400% of the FPL (see Appendix Table A1) (to access the Appendix, click on the Details tab of the article online), and (3) for the last 6 months of 2021 expanded CSR silver 94 eligibility for individuals who received unemployment insurance (UI).^1^

While the effects of the ARPA on overall Marketplace enrollment is now well documented, much less is known about how the ARPA—and the temporary expansion and the automatic redetermination of CSR silver 94 eligibility for UI recipients—affected enrollees’ premiums and out-of-pocket expenses.^2-4^

We seek to fill this gap by analyzing an intervention in California's ACA Marketplace that targeted enrollees who reported receiving UI and were in silver-tier plans. California's ACA Marketplace redetermined eligibility for 79 645 UI recipients already in silver-tier plans in June 2021 and automatically re-enrolled them in a CSR silver 94 plan.^5^ Evidence from this intervention can inform state and federal policymakers seeking to understand both the impact of ARPA programs and effective strategies for quickly connecting people to benefits for which they are eligible, especially when they are already engaging with safety-net programs.^6^

## Data and methods

### Study population

The intervention was administered in California's state-based ACA Marketplace, Covered California, during the 2021 coverage year among 79 645 individuals who reported receiving UI for at least 1 week in 2021 and were enrolled in a silver-tier plan. Prior to the intervention in June 2021, 14 166 enrollees were already in CSR silver 94 plans, while the remaining 65 479 enrollees were in silver 70, 73, or 87 plans.

### Intervention

In the third week of June, Covered California implemented an automatic eligibility redetermination for all 79 645 individuals. This automated process enabled individuals claiming UI in 2021 to qualify for maximum subsidies usually reserved for individuals with incomes of 138% of the FPL. The same automatic process re-enrolled individuals in silver 70, 73, or 87 plans into silver 94 plans with their existing insurer and network effective July 1, 2021. These individuals then received a notice explaining that Covered California re-ran their eligibility to apply additional financial help as part of the ARPA's UI provision and included information about cost-sharing reductions to lower costs when using medical care (see Appendix Figure A1).

Since all enrolled individuals eligible for the enhanced ARPA subsidies were included in the automatic redetermination, we used exposure intensity as the basis for creating treatment and control groups. Those who were in CSR silver 94 plans prior to the redetermination were considered minimally exposed and thus served as the control group, while those who were automatically moved into a CSR silver 94 plan were maximally exposed and thus served as the treatment group.

### Data sources

For our analysis, we used administrative data from Covered California for 2 periods: a pre-intervention period spanning January–June 2021 and a post-intervention period covering July–December 2021. These data provide individual-level demographic, eligibility, and enrollment details along with out-of-pocket spending.

### Outcomes

Drawing on the data above, our study includes 2 outcomes of interest: net-of-subsidy premium and out-of-pocket costs, both at the individual level for pre- and post-intervention periods. The amounts are aggregated and represent the total amount for each 6-month period.

### Statistical analysis

We estimated the effect of the ARPA's UI provision using a difference-in-differences (DID) design with 2 periods, which compares pre-post changes in outcomes among the treatment group while accounting for underlying trends in outcomes in the control group. The DID approach assumes parallel trends in average outcomes for the control and treatment groups in the absence of the intervention; however, we do not have multiple pre-intervention periods for both outcomes and thus are unable to test that assumption (see Appendix Figure A2).

### Stratification variables

To assess how the automatic redetermination under the ARPA's UI provision affected various subpopulations, we estimated additional DID regressions, stratifying the data by baseline silver plan variant, self-reported race and ethnicity, chronic condition (defined as a diagnosis of diabetes, HIV, asthma, or hypertension), and age bracket.

### Limitations

Our study has several limitations. First, the ARPA's UI provision was only in effect for the last 6 months of 2021. And it is likely the case that not all enrollees understood that Covered California moved them to a silver 94 plan with lower costs when accessing care. In tandem, the short duration of the policy coupled with limited awareness could dilute the effect of the intervention.

Second, our data are limited to a single state and may not reflect the effects of ARPA's UI provision that other states experience if those states pursued different implementation strategies. Finally, we did not evaluate the changes in demand for health care services following the change in cost-sharing reduction eligibility, which could be pursued in future research.

## Results

The sample included 79 645 individuals who reported UI and were in silver-tier plans prior to and after the automatic redetermination, bringing the total number of observations to 159 290. As noted above, the treatment group consisted of 65 479 individuals who were moved into CSR silver 94 plans from July to December 2021 and the control group consisted of 14 166 already in CSR silver 94 plans prior to the automatic redetermination. Compared with individuals in non–silver 94 plans, individuals in silver 94 plans prior to the redetermination had lower incomes and were more likely to identify as Asian, less likely to identify as White, and less likely to list English as their preferred written language. In addition, individuals in silver 94 plans spent less on premiums and out-of-pocket expenses than those in non–silver 94 plans (see Appendix Table A2).

### Effects of ARPA UI provision

**Figure 1.**
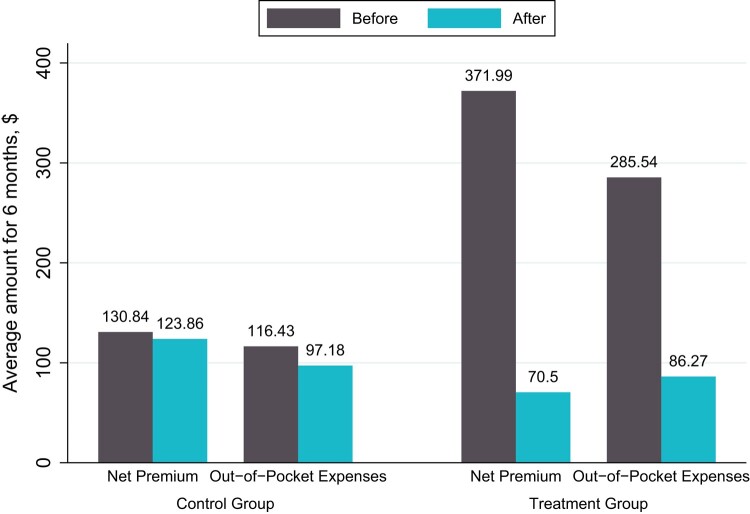
Effects of ARPA UI provision on net premium and out-of-pocket expenses savings. Source: Authors’ analysis of administrative data from Covered California, 2021. “Before” refers to the pre-intervention period from January 2021 to June 2021. “After” refers to the post-intervention period from July 2021 to December 2021. The control group consists of individuals who were in CSR silver 94 plans prior to the intervention, and the treatment group consists of individuals who were automatically moved to CSR silver 94 plans for the last 6 months of 2021. The figure plots coefficients from difference-in-differences regression. Abbreviations: ARPA, American Rescue Plan Act; CSR, cost-sharing reduction; UI, unemployment insurance.

We found that automatically moving individuals into CSR silver 94 coverage for the last 6 months of 2021 was associated with a statistically significant $295 decrease in net premiums and a statistically significant $180 decrease in out-of-pocket expenses (Figure 1). Together, this amounts to a total average savings of $475.

All subgroups experienced statistically significant decreases in premiums and out-of-pocket expenses (Table 1). Because the ACA's subsidies are income-based, and many of the lowest-income enrollees already qualify for very low premiums and out-of-pocket costs, premium savings were largest for middle-income individuals in Silver 70 plans prior to the redetermination ($968). These middle-income individuals in silver 70 and silver 73 plans prior to the redetermination also reaped larger out-of-pocket savings ($261 and $265, respectively) by being moved to more generous CSR silver 94 coverage. The largest out-of-pocket savings were among individuals with a chronic condition ($392).

**Table 1.** Effects of ARPA UI provision on net premium and out-of-pocket costs by subgroup.

	No. of observations	DID net premium, point estimate (SE)	DID OOP, point estimate (SE)
Plan type at baseline			
Silver 70	59 178	−967.92*** (8.53)	−260.83*** (10.33)
Silver 73	49 784	−221.70*** (6.04)	−264.65*** (11.39)
Silver 87	106 992	−50.29*** (4.15)	−125.26*** (5.55)
Race and ethnicity of enrollee			
Asian	29 474	−271.21*** (8.34)	−117.31*** (9.35)
Black	5346	−215.59*** (20.94)	−211.85*** (33.61)
Latino	40 136	−214.35*** (6.61)	−174.79*** (10.17)
White	44 874	−360.61*** (11.41)	−199.45*** (12.98)
Other/unknown	39 460	−325.47*** (9.93)	−193.41*** (11.52)
Chronic condition			
Chronic condition	22 728	−284.59*** (13.90)	−391.56*** (19.79)
No chronic condition	136 562	−296.12*** (4.77)	−143.23*** (5.41)
Age of enrollee			
Under 30 y	28 980	−234.35*** (6.63)	−109.543*** (12.020)
30 to 50 y	60 812	−286.18*** (6.29)	−148.254*** (8.793)
Over 50 y	69 498	−324.20*** (8.66)	−233.624*** (8.331)

Abbreviations: ARPA, American Rescue Plan Act; DID, difference-in-differences; OOP, out-of-pocket; UI, unemployment insurance.

Source: Authors’ analysis of administrative data from Covered California, 2021. Robust standard errors are in parentheses. Chronic condition includes anyone diagnosed with diabetes, HIV, asthma, or hypertension prior to the automatic redetermination. Race and ethnicity are self-reported by enrollees on their application. “Other” refers to all other race and ethnic groups (>10 reported) and “unknown” if enrollees choose not to report their race and ethnicity. ***P < .001.

## Discussion and conclusion

The ARPA temporarily expanded premium subsidies and cost-sharing reduction silver 94 plans—the most generous coverage option available in the ACA Marketplaces—to UI recipients for the last 6 months of 2021. Our study examined the effects of automatic redetermination under this policy change using a DID design with detailed individual-level administrative data. We found that the ARPA's UI provision significantly increased premium and out-of-pocket savings for individuals who were automatically moved to CSR silver 94 coverage in California's ACA Marketplace. Savings were larger for middle-income individuals (as a percentage of FPL), given that lower-income individuals were already benefitting from substantial premium and out-of-pocket subsidies. Further, our results indicate that individuals with a chronic condition saved larger amounts than those without, indicating that policies that lower out-of-pocket costs benefit those with the greatest health needs.^7^

Our estimates are consistent with prior research on the effects of switching to a CSR silver plan but are a result of a stronger intervention. Prior studies used informational nudges to induce low-income households to switch into CSR plans, but take-up was modest, with more than three-quarters of the study group remaining in less generous plans after the nudges.^8,9^ In the setting for this study, California's ACA Marketplace automatically moved all eligible individuals into richer coverage at a lower price to ensure they received enhanced benefits while they were available.

Overall, evidence from this intervention demonstrates the material impact of a novel policy that connected consumers experiencing job loss with enhanced premium and cost-sharing assistance during the COVID-19 pandemic. By using existing administrative records to determine eligibility and subsequently defaulting individuals into improved health care affordability benefits, our intervention can inform state and federal policymakers on 1 potential approach for reducing administrative burdens and automating the delivery of safety-net benefits.

## Supplementary Material

qxae054_Supplementary_Data
