# Supplementary material for: Automating benefits delivery: lowering health insurance costs for unemployment insurance recipients
Source: Health Aff Sch. 2024 May 2;2(5):qxae054. doi: 10.1093/haschl/qxae054 (PMC11095557; doi:10.1093/haschl/qxae054)
Supplement: qxae054_Supplementary_Data [file qxae054_supplementary_data.zip › Online_Appendix_20240423.docx]

Online Appendix to Automating Benefits Delivery: Lowering Health Insurance Premiums and Out-of-pocket Costs for Unemployment Insurance Recipients

Contents

[**Required premium contribution percentage by Federal Poverty Level (FPL), before and after ARPA.** 2](#_Toc163658231)

[Table A1 2](#_Toc163658232)

[**Intervention Material** 3](#_Toc163658233)

[Figure A1: Eligibility Redetermination Notice Letter 3](#_Toc163658234)

[**Table A2: Characteristics of Covered California enrollees in silver plans who reported UI in 2021 at baseline** 5](#_Toc163658235)

[**Figure A2: Trends in monthly net premium in pre-intervention period, by treatment group** 6](#_Toc163658236)

[**Source and definition of out-of-pocket measure used in the study** 7](#_Toc163658237)

# **Required premium contribution percentage by Federal Poverty Level (FPL), before and after ARPA.**

As noted in the main text, the American Rescue Plan Act (ARPA) enhanced the ACA’s subsidy structure by lowering the required contribution percentage for all ACA enrollees. For those with incomes above 400% of the federal poverty level (FPL), the ARPA also capped premium contributions at 8.5% of income.

## Table A1

| Percentage of income contributed for premiums, based on household FPL (2021) | | |
| --- | --- | --- |
| Household FPL Percentage | Required premium contribution under ACA | Required premium contribution under ARPA |
| Below 138% FPL | 2.07% | 0% |
| 138%-150% FPL^1^ | 3.1 - 4.14% | 0% |
| 150-200% FPL | 4.14 - 6.52% | 0 - 2% |
| 200-250% FPL | 6.52 - 8.33% | 2 - 4% |
| 250-300% FPL | 8.33 - 9.83% | 4 - 6% |
| 300-400% | 9.83% | 6 - 8.5% |
| Above 400+ FPL | - | 8.5% |

Source: <https://www.irs.gov/pub/irs-drop/rp-20-36.pdf>. <https://www.irs.gov/irb/2021-35_IRB#REV-PROC-2021-36>.

Note: ^1^ California uses 138% of the federal poverty level as upper bound for Medicaid Eligibility.

# **Intervention Material**

## Figure A1: Eligibility Redetermination Notice Letter

**Table A2: Characteristics of Covered California enrollees in silver plans who reported UI in 2021 in pre-intervention period.**

|  | January-June 2021 | |
| --- | --- | --- |
|  | Individuals in CSR silver 94 plans (n = 14,166) | Individuals in silver plans (n = 65,479) |
| Income as a percent of FPL | 141% | 222% |
| Age | 44 | 45 |
| Language preference of enrollee |  |  |
| English | 80% | 83% |
| Spanish | 11% | 11% |
| Race and ethnicity of enrollee |  |  |
| Asian | 25% | 17% |
| Black | 4% | 3% |
| Latino | 25% | 25% |
| White | 23% | 29% |
| Other/Unknown | 24% | 25% |
| Outcomes |  |  |
| Net premium | $131 | $372 |
| Out-of-pocket spending | $116 | $286 |

Source: Authors’ analysis of administrative data from Covered California, 2021. Race and ethnicity are self-reported by enrollees on their application. Other or Unknown refers to all other race and ethnicities groups (more than 10 reported) and to unknown group if enrollees choose not to report their race and ethnicity. Outcomes are total amounts for six-month pre-intervention period averaged by group.

**Figure A2: Trends in monthly net premium in pre-intervention period, by treatment group**

The difference-in-difference (DID) design assumes parallel trends in average outcomes in the absence of the intervention. Our data, however, contains only one pre-treatment period, thus, precluding us from performing a pre-trend test.

Using a different dataset, we perform an informal test of the DID’s main identifying assumption, plotting the average monthly net-of-subsidy premium in the pre-treatment period from January to June 2021 for the enrollees not in silver 94 plans (treatment group) and enrollees in silver 94 plans (control group). In the figure below, we can see that the two trend lines are similar, providing suggestive evidence that the parallel paths identifying assumption is plausible.


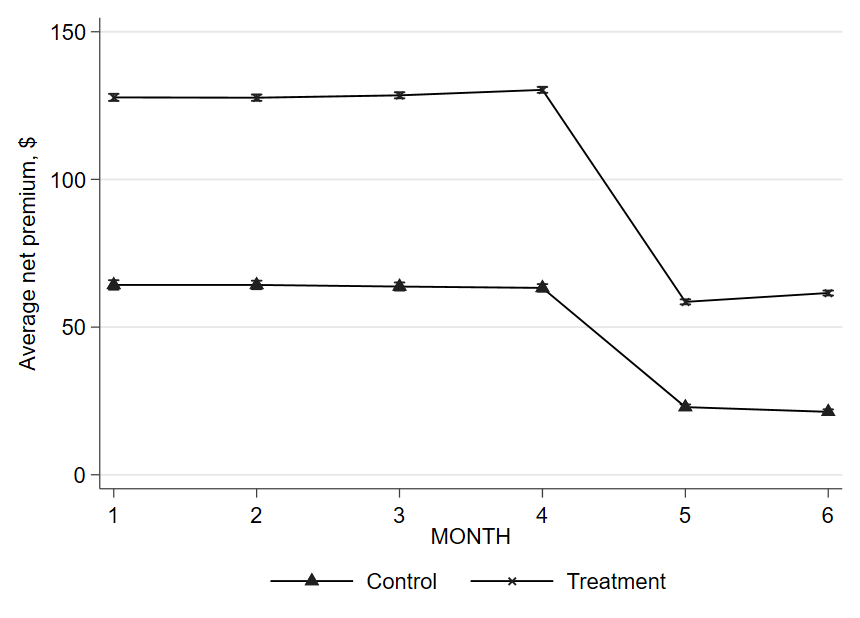


**Source and definition of out-of-pocket measure used in the study.**

**Health Care Utilization Data**: Covered California requires all contracted health plan issuers to submit claims and utilization data as part of AB929.^^[[1]](#footnote-1)^^

Measure used in the study is defined as follows:

**Out-of-pocket expenses** is the amount paid out-of-pocket by the member for facility, professional and prescription drug services. This generally include coinsurance, copayment and deductible amounts.

1. Assembly Bill 929 Paragraph 100503.7 (b) (1) states: “A qualified health plan shall provide data on enrollees to the Exchange in a form, manner, and frequency specified by the Exchange.” For additional details about legislation see https://leginfo.legislature.ca.gov/faces/billNavClient.xhtml?bill_id=201920200AB929 [↑](#footnote-ref-1)
